# Systematic Assessment of Tumor Purity and Its Clinical Implications

**DOI:** 10.1200/PO.20.00016

**Published:** 2020-09-04

**Authors:** Syed Haider, Svitlana Tyekucheva, Davide Prandi, Natalie S. Fox, Jaeil Ahn, Andrew Wei Xu, Angeliki Pantazi, Peter J. Park, Peter W. Laird, Chris Sander, Wenyi Wang, Francesca Demichelis, Massimo Loda, Paul C. Boutros, The Cancer Genome Atlas Research Network

**Affiliations:** ^1^ Ontario Institute for Cancer Research, Toronto, Ontario, Canada; ^2^ The Breast Cancer Now Toby Robins Research Centre, The Institute of Cancer Research, London, United Kingdom; ^3^ Department of Data Sciences, Dana-Farber Cancer Institute, Boston, MA; ^4^ Department of Biostatistics, Harvard T.H. Chan School of Public Health, Boston, MA; ^5^ Department of Cellular, Computational and Integrative Biology, University of Trento, Trento, Italy; ^6^ Department of Medical Biophysics, University of Toronto, Toronto, Ontario, Canada; ^7^ Department of Biostatistics, Bioinformatics and Biomathematics, Georgetown University Medical Center, Washington, DC; ^8^ Department of Biomedical Informatics, Harvard Medical School, Boston, MA; ^9^ Brigham and Women’s Hospital, Boston, MA; ^10^ Van Andel Research Institute, Grand Rapids, MI; ^11^ cBio Center, Dana-Farber Cancer Institute, Boston, MA; ^12^ Department of Cell Biology, Harvard Medical School, Boston, MA; ^13^ The University of Texas MD Anderson Cancer Center Department of Bioinformatics and Computational Biology, Houston; ^14^ Englander Institute for Precision Medicine, New York Presbyterian Hospital, Weill Cornell Medicine, New York, NY; ^15^ Department of Pathology, Weill Medical College of Cornell University, New York, NY; ^16^ Department of Oncologic Pathology, Dana-Farber Cancer Institute, Boston, MA; ^17^ Department of Human Genetics, University of California, Los Angeles, CA; ^18^ Department of Urology, University of California, Los Angeles, CA; ^19^ Jonsson Comprehensive Cancer Center, University of California, Los Angeles, CA; ^20^ Institute for Precision Health, University of California, Los Angeles, CA

## Abstract

**PURPOSE:**

The tumor microenvironment is complex, comprising heterogeneous cellular populations. As molecular profiles are frequently generated using bulk tissue sections, they represent an admixture of multiple cell types (including immune, stromal, and cancer cells) interacting with each other. Therefore, these molecular profiles are confounded by signals emanating from many cell types. Accurate assessment of residual cancer cell fraction is crucial for parameterization and interpretation of genomic analyses, as well as for accurately interpreting the clinical properties of the tumor.

**MATERIALS AND METHODS:**

To benchmark cancer cell fraction estimation methods, 10 estimators were applied to a clinical cohort of 333 patients with prostate cancer. These methods include gold-standard multiobserver pathology estimates, as well as estimates inferred from genome, epigenome, and transcriptome data. In addition, two methods based on genomic and transcriptomic profiles were used to quantify tumor purity in 4,497 tumors across 12 cancer types. Bulk mRNA and microRNA profiles were subject to in silico deconvolution to estimate cancer cell–specific mRNA and microRNA profiles.

**RESULTS:**

We present a systematic comparison of 10 tumor purity estimation methods on a cohort of 333 prostate tumors. We quantify variation among purity estimation methods and demonstrate how this influences interpretation of clinico-genomic analyses. Our data show poor concordance between pathologic and molecular purity estimates, necessitating caution when interpreting molecular results. Limited concordance between DNA- and mRNA-derived purity estimates remained a general pan-cancer phenomenon when tested in an additional 4,497 tumors spanning 12 cancer types.

**CONCLUSION:**

The choice of tumor purity estimation method may have a profound impact on the interpretation of genomic assays. Taken together, these data highlight the need for improved assessment of tumor purity and quantitation of its influences on the molecular hallmarks of cancers.

## INTRODUCTION

The tumor microenvironment represents an admixture of multiple cell types and complex interactions between bona fide cancer cells and surrounding stromal and immune cells.^1^ Because a majority of high-throughput experiments are performed on bulk tissue samples, the resulting signal is usually confounded by nonmalignant tumor-adjacent cells (TACs). Variable tumor content and variable TAC composition can impinge upon interpretations of molecular data and subsequent clinical decisions.^2-4^ To delineate true residual signal representing individual cell populations, it is crucial to accurately estimate tumor purity. Tumor purity represents the fraction of cancer cells in a tumor and can be estimated either by expert pathologists reviewing tumor sections^5^ or in silico (using epigenomic, genomic, or transcriptomic profiles).^6^ Pathologic estimates can be inconsistent^5^ and pragmatically may not always represent the region of tumor that is subject to molecular profiling. Although in silico estimates could circumvent these problems, it remains unclear to what extent these estimates vary across purity calling methods and with the underlying type of biomolecule (eg, DNA *v* RNA). Previous studies have quantified the pan-cancer purity landscape^2,7^ and compared a panel of tools for estimating tumor purity.^6^ However, systematic benchmarking of in silico tumor purity against matched pathologic estimates and its association with multimodal clinico-genomic profiles remains to be elucidated. Herein, we present systematic benchmarking of 10 purity estimation methods using DNA, mRNA, and microRNA (miRNA) profiles in a 333-patient clinically-coherent cohort^8^ with matched multiobserver pathologic estimates of purity. We then quantify how molecular correlates of tumor purity can skew clinico-genomic interpretations as the result of variable estimates of cancer cell fraction. Last, we demonstrate a comparison between the purity estimates inferred from most commonly used molecular profiles (DNA and RNA) across 12 additional cancer types.

**Context**

**Key Objective**
Tumor cell fraction (also called tumor purity) is routinely estimated by expert pathologists. Genome-wide molecular assays have led to active development of in silico algorithms for estimating tumor purity. To determine the context specificity of these algorithms, we compared tumor purity estimates from multiobserver pathology to those from multiple algorithms working on different biomolecules (eg, DNA, RNA).
**Knowledge Generated**
Tumor purity estimates from in silico tools varied significantly from pathology estimates. In silico purity estimates were biased by the biomolecule type. We recommend parameterizing genomic analyses with tumor purity estimated from the matched molecular analyte being analyzed.
**Relevance**
Tumor purity is a key criterion for sample inclusion in clinico-genomic studies and subsequent interpretation of molecular results. Computational tools often require purity estimates; we show that these are influenced by the selected purity estimator. Both molecularly driven clinical trials, as well as therapeutic and theranostic decisions, may be affected by these choices.

## MATERIALS AND METHODS

### Methods for Purity Estimation

Prostate cancer purity estimates were generated by multiple pathologists using top and bottom slides, as previously described.^8^ In silico estimates were generated using a panel of previously published DNA-, mRNA-, and microRNA-based methods^11-17^ (ASCAT v2.1, CLONET v1.0.0, OncoSNP v3.0.1, ISOpure v1.3) and two additional unpublished methods (LEUC, genomic methylation signature of leukocytes [as previously described in ref^13^] and INTEGER, a low-pass DNA sequencing–based method that was run on a subset of cohort (115 samples, of which 107 were present in the data freeze used for this study). INTEGER infers purity, ploidy, and subclonality from paired tumor and normal samples using the following principles: (1) models the relationship between the observed allelic frequencies and the underlying copy number changes, and the possible existences and impacts of multiple subclones that may often mislead inferences if not explicitly modeled; (2) simultaneous statistic inference on the basis of both copy number changes and major allelic frequencies; (3) restoration of information lost as a result of the guanine-cytosine content and actual sizes of each library insert and other specific biases of each genomic location; (4) avoid making inferences when the signal-to-noise ratio is not ideal because of technical artifacts; (5) an explicit modeling of whole-genome duplication events and whole-chromosome duplication events, which are common in cancer genomics and have huge impacts on the accurate inference of purity and ploidy; and (6) high statistical power with the possibility to make reliable inferences on low-pass genomic data (as low as 0.5× sequencing depths).

Three microRNA samples were missing from The Cancer Genome Atlas data repository and are therefore not included in this study.

The Cancer Genome Atlas pan-cancer purity estimates were generated using processed RNA-Seq data (for ISOpure) downloaded from https://gdac.broadinstitute.org/ (download version 2015) and SNP6 array level-1 data (for ASCAT) downloaded from GDC data portal.

### Consensus Pathology, DNA, and mRNA Purity Estimates

Multiobserver pathology reviews yielded purity ranges,^8^ which were further collapsed into single-point estimates using the median value of purity range in deciles. DNA (ABSOLUTE, ASCAT, CLONET, INTEGER, OncoSNP)– and mRNA (DeMix and ISOpure-R)–based purity estimates were aggregated using median DNA and mRNA estimates, respectively.

### Availability of Data and Materials

All processed data are available either in the Data Supplement or uploaded to DOI: 10.5281/zenodo.3349831 as specified in the Data Supplement. TCGA prostate adenocarcinoma study data are available in the original publication.^8^

### Recurrently Altered Genes Panel, Androgen Receptor Signature, Percent Genome Altered, SNVs, and Clinical Covariables

These data sets were reused from the original publication.^8^

### Data Analysis and Visualizations

All data analyses were performed using R statistical programming language (v3.4.4). All statistical tests were two sided. Visualizations were created using R package BoutrosLab.plotting.general (v5.9.2).^28^

### Ethics Approval and Consent to Participate

Tissue contributing sites followed appropriate consent documentation and approved submission of cases to The Cancer Genome Atlas, as detailed in the original publication.^8^

## RESULTS

**FIG 1.**
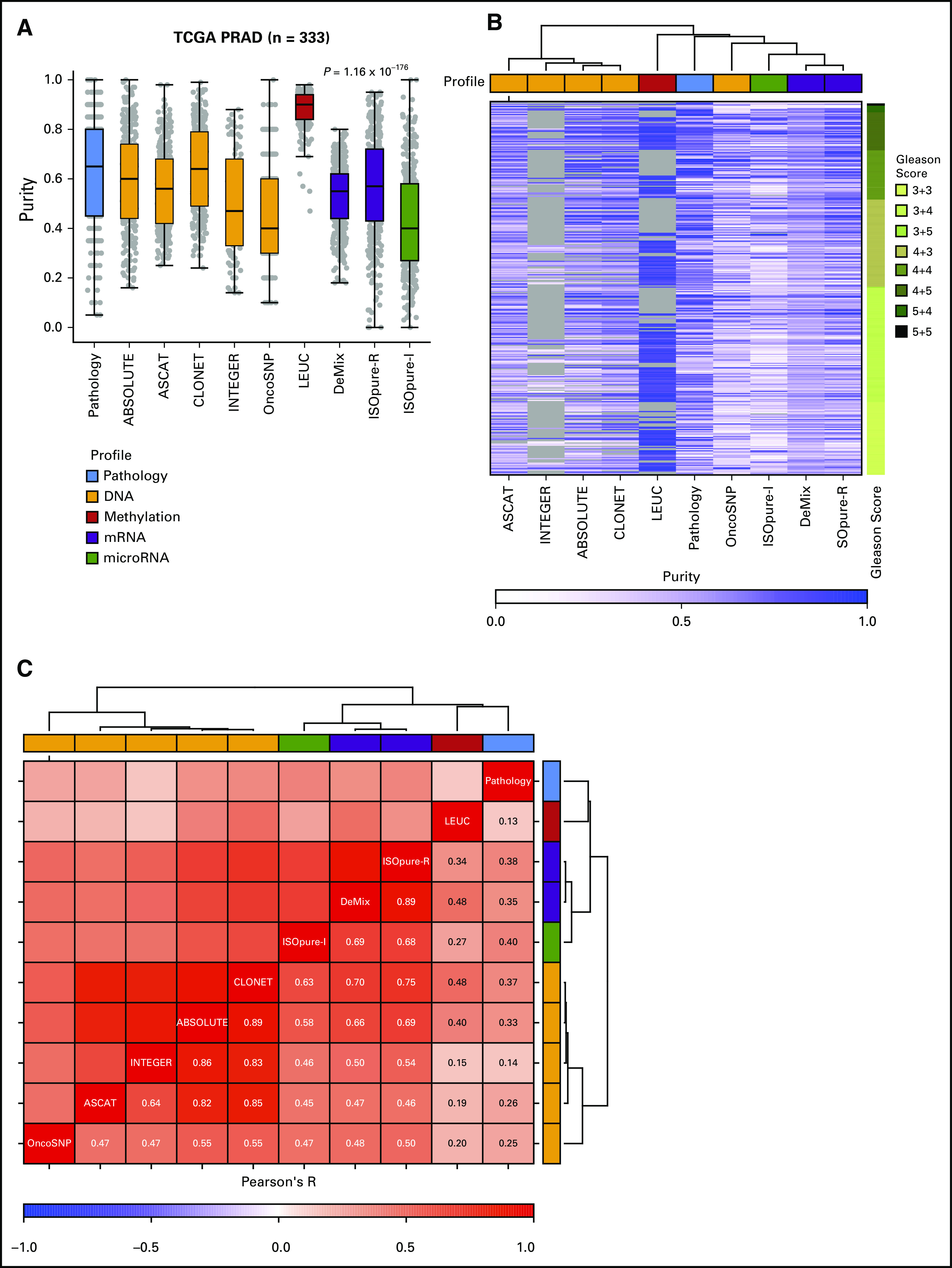
Purity landscape in The Cancer Genome Atlas (TCGA) prostate cancer cohort (PRAD). (A) Distribution of TCGA prostate tumor purity estimates (n = 333) using in silico methods and consolidated multiobserver pathology reviews; (B) Patient-wise purity estimates grouped by Gleason score. Gray represents missing data, including both failed estimates and missing molecular profiles (see Methods for details). Columns were clustered using Ward hierarchical clustering method. Data from INTEGER were available for 107 samples using the low-pass DNA sequencing data; (C) Pearson correlation between purity estimates inferred using in silico methods and pathology reviews. Rows and columns were clustered using Ward hierarchical clustering method.

Prostate cancer presents complex intra- and interpatient heterogeneity. It is an ideal model to study heterogeneity because of frequent surgical management via radical prostatectomy of the whole gland, allowing spatio-genomic studies.^9,10^ We collated pathologic, molecular, and clinical data sets from The Cancer Genome Atlas’ (TCGA) prostate marker study, which comprised 333 patients.^8^ Purity estimates from multiple pathologists were consolidated, resulting in point estimates as previously described^8^ (see Methods). For a subset of cases, both top and bottom tissue block slides (with sections acquired for molecular analysis in between these) were assessed by multiple pathologists, demonstrating moderate correlation between pathologists (top sections: Pearson’s R = 0.64, *P* = 6.23 × 10^−7^; bottom sections: Pearson’s R = 0.53, *P* = 8.93 × 10^−3^; Data Supplement Fig 1A-B). A similar trend was observed between the pathology estimates of top and bottom sections (Pearson’s R = 0.59, *P* = 2.03 × 10^−12^; Data Supplement Fig 1C), highlighting potential influence of spatial heterogeneity. In silico estimates of tumor purity were generated using nine methods^11-18^ that leverage DNA (methylation or copy number data), mRNA, or miRNA profiles (Data Supplement Tables 1 and 2; Methods). These purity estimates demonstrated considerable intermethod variation (*P*_analysis of variance_ = 1.16 × 10^−176^; Fig 1A). Of note, LEUC estimates on the basis of DNA methylation data were right skewed, with a median purity of 0.9 (Δ_LEUC-Other_ = 0.33, *P* = 1.44 × 10^−95^, Wilcoxon rank sum test). This is expected because they represent an upper bound of tumor content by estimating the percentage of leukocytes in a specimen.

Among the panel of methods assessed, five failed to estimate purity on the complete data set (percentage missing: ASCAT = 4.8%, CLONET = 12.9%, ABSOLUTE = 14.1%, INTEGER = 16.8%, LEUC = 40.5%). Interestingly, all these methods were based on DNA profiles (genomic or epigenomic), suggesting intrinsic limitations in estimating tumor purity from DNA-based assays in this setting. These limitations could be explained by the DNA profile itself, because samples with failed purity estimates exhibited quiet genomes with low numbers of somatic single nucleotide variants (SNVs; Data Supplement Fig 2A-B). We tested whether these failed samples were considered low-purity samples by pathology and RNA-based methods. Pathology calls did not show clear evidence of low purity; however, RNA-based methods predicted a trend toward low purity for a subset of samples (Data Supplement Fig 2C). Some of these failed samples may thus truly have low tumor cellularity. However, it is probable that some may also represent quiet cancer genomes, which are now increasingly recognized as a real phenomenon, particularly in prostate cancer.^8,19^

Inspection of the complete sample set revealed no association with histologic heterogeneity (rationalized as Gleason score^10^; Fig 1B, Data Supplement Fig 3A-B). Tumor purity estimates across methods strongly clustered with the type of molecular profile used to generate them (Fig 1B). DNA copy number–based assays showed strong correlation among themselves (Pearson’s R between each pair of methods = 0.47 to 0.89), and RNA-based methods exhibited similar strong intraprofile correlation (Pearson’s R between each pair of methods = 0.68 to 0.89; Fig 1C). DNA methylation–based LEUC estimates showed weak/moderate correlation with other DNA- and RNA-based methods (Pearson’s R between LEUC and other methods = 0.15 to 0.48; Fig 1C). Surprisingly, pathology estimates were weakly correlated with the other nine methods (Pearson’s R between pathology and other methods = 0.13 to 0.40; Fig 1C, Data Supplement Fig 4A). This raised concerns about the appropriateness of pathology estimates in parameterizing bioinformatics tools that analyze DNA or RNA profiles. Moreover, correlation between in silico callers and pathology estimates of top and bottom sections separately remained weak (Pearson’s R = 0.04 to 0.32, Data Supplement Fig 4B). Hence, we preclude spatial heterogeneity as the primary factor underlying this lack of concordance.

**FIG 2.**
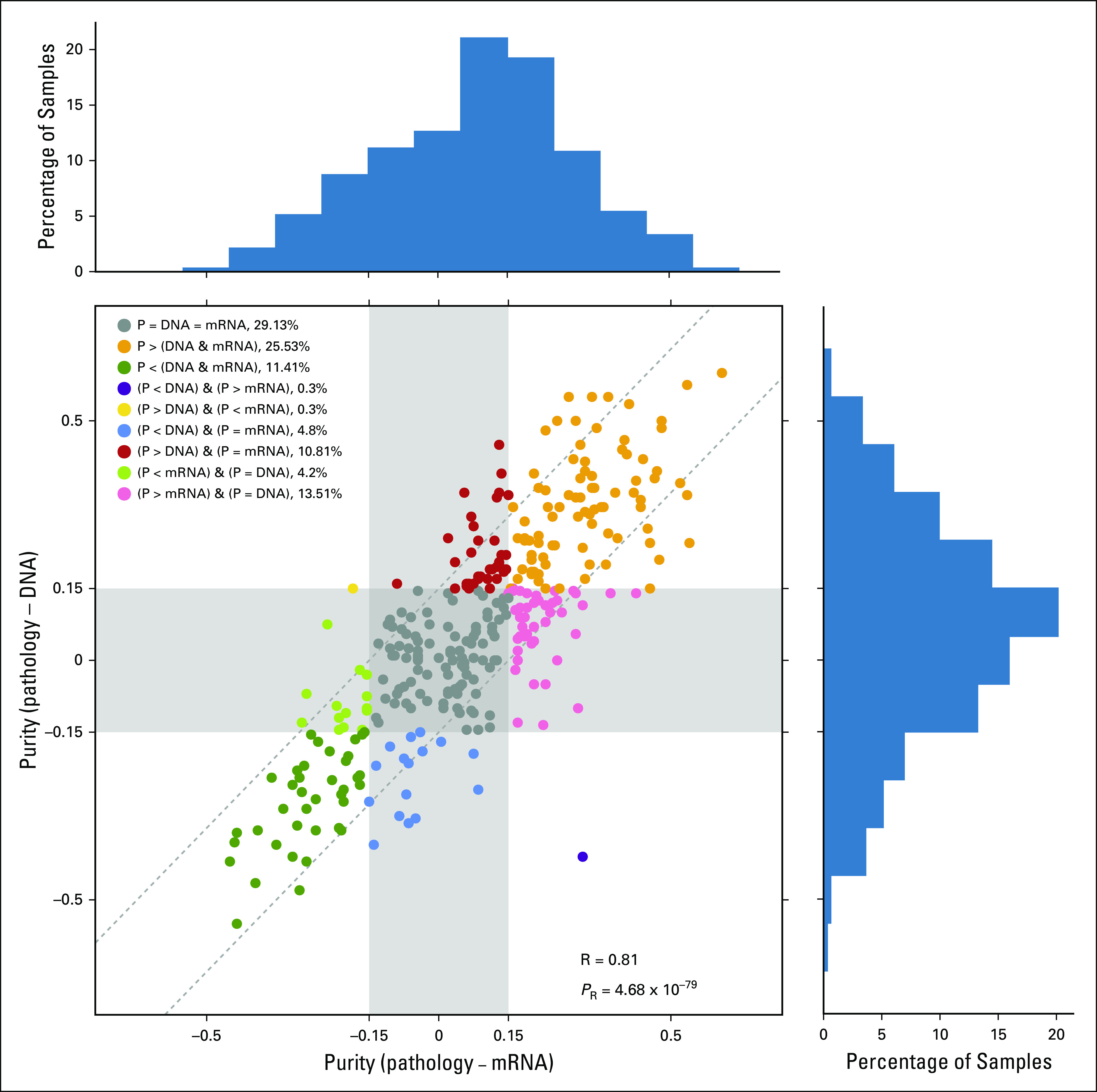
Deviation of pathologist-inferred tumor purity from in silico estimates. Difference between pathology estimates of tumor purity and in silico estimates from DNA and mRNA abundance profiles. P, pathology estimates; R, Pearson’s correlation coefficient; *P*_R_, statistical significance of observed correlation.

These data highlight that variation and error profiles among the intraplatform estimates are probably correlated and suffer from similar intrinsic limitations, independent of the specific algorithm used. Therefore, we created consensus DNA and mRNA purity estimates using the median for each class of methods, hereafter referred to as DNA and mRNA estimates (see Methods). The differences between pathology estimates and either DNA or mRNA estimates were strongly correlated (Pearson’s R = 0.81, *P* = 4.68 × 10^−79^; Fig 2), with 29.13% of cases demonstrating agreement (within 15% purity of each other). Samples that had agreement in DNA and mRNA estimates were significantly more likely to underestimate (UE) than overestimate (OE) purity relative to pathology estimates (UE = 25.5% of cases, OE = 11.4%, *P*_Binomial_ = 2.72 × 10^−5^). This trend persisted when DNA and mRNA estimates were compared with pathology independently (DNA: Δ_UE−OE_ = 20.13%, mRNA: Δ_UE−OE_ = 23.43%). Of the DNA- and mRNA-based estimates, only two samples displayed discordant directions of effect relative to pathologic estimates (purple and yellow dots in Fig 2), highlighting overall similarity in error profiles of the underlying biomolecules.

**FIG 3.**
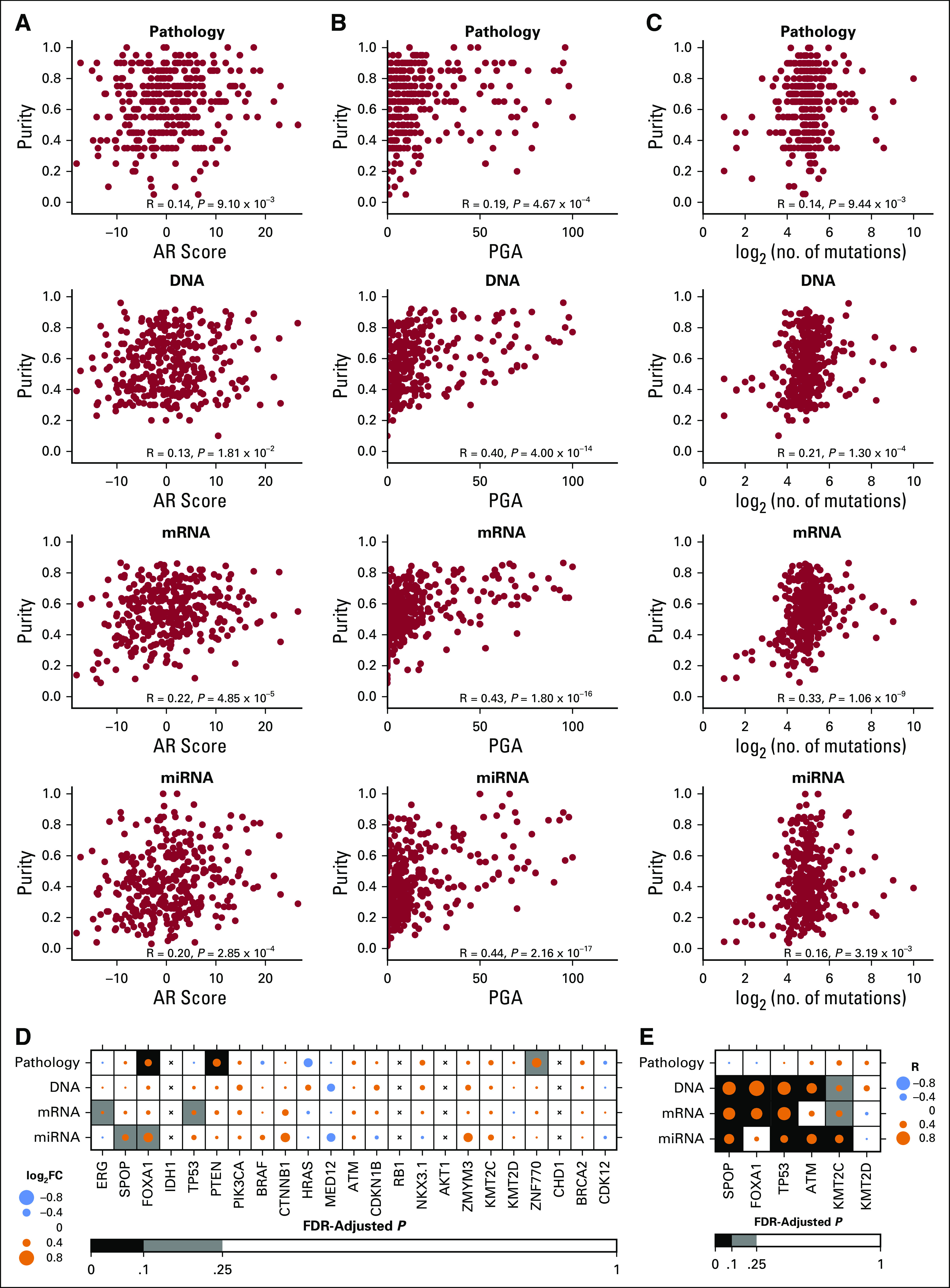
Molecular correlates of tumor purity. Genomic correlates of tumor purity as summarized using androgen receptor (AR) signature score (A), percent genome altered ([PGA], B), and mutation burden (C). Correlation statistic was estimated using Pearson correlation. (D) Purity estimates stratified by prostate cancer–specific driver mutations and *ERG* fusions. log_2_FC represents difference in mean purity (log_2_ scale) between mutant and wild-type samples (ERG represents *ERG* fusions). Statistical significance was estimated using Wilcoxon rank sum test, and *P* values were adjusted for multiple comparisons using the Benjamini–Hochberg method. Statistical tests were performed for genes with more than three mutant samples. Therefore, *IDH1*, *RB1*, *AKT1*, and *CHD1* (displayed with “x”) were deemed inappropriate for statistical testing. (E) Correlation between purity estimates and variant allele frequency of mutant samples. Correlation statistic was estimated using Pearson correlation, and *P* values were adjusted for multiple comparisons using the Benjamini–Hochberg method. For reliable correlation estimates, genes (in panel 3D) with more than 10 mutant samples were considered for estimating correlation with tumor purity. FDR, false discovery rate; miRNA, microRNA.

Next, we assessed whether the key transcriptional and genomic biomarkers that underpin prostate cancer biology are dependent on tumor purity. The activity of androgen receptor transcriptional targets (AR Score) showed no association with pathologic or DNA-based methods while demonstrating a weak association with mRNA- and miRNA-derived purity estimates (Pearson’s R = 0.20 to 0.22, *P* < .001; Fig 3A). Genomic instability (percent genome altered), a strong predictor of disease aggressiveness,^20^ was weakly associated with pathologic estimates of purity (Pearson’s R = 0.19) and moderately correlated with purity derived from DNA, mRNA, and miRNA profiles (Pearson’s R = 0.40 to 0.44, *P* < .001; Fig 3B). SNV mutation burden was weakly associated with DNA- and mRNA-based purity estimates (Pearson’s R = 0.21 to 0.33, *P* < .001; Fig 3C). To further delineate the relationship between tumor purity and somatic mutations, we stratified purity estimates by the mutation status of a panel of recurrently altered genes in prostate cancer.^8^ Tumor purity determined by at least one profile was associated with six genes, including *ERG* fusions and *SPOP*, *FOXA1*, and *TP53* point mutations (false discovery rate [FDR]–adjusted *P* < .25, Wilcoxon rank sum test; Fig 3D, Data Supplement Table 3). For these six genes, tumor purity was moderately higher in mutant samples.

To characterize this association between driver gene status and tumor purity, we evaluated the associations between tumor purity and the variant allele frequency (VAF) in samples carrying mutations (Fig 3E). Tumor purity inferred by at least one of the DNA and RNA analytes was positively correlated with the VAF, in particular, demonstrating strong associations with *SPOP*, *FOXA1*, *TP53*, *ATM*, and *KMT2C* (FDR-adjusted *P* < .1). However, pathology estimates of tumor purity were unable to accurately capture the VAF of these recurrently altered genes.

**FIG 4.**
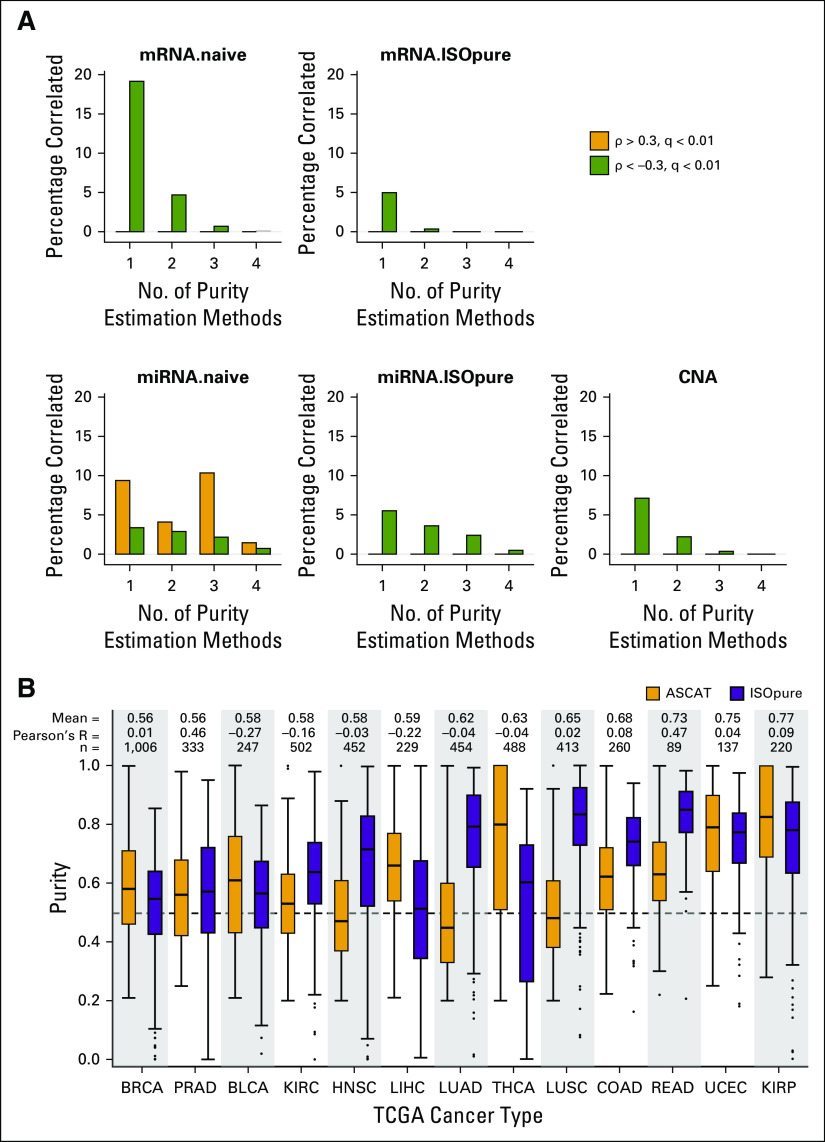
Deconvolved prostate cancer profiles, and DNA- and mRNA-derived purity estimates across The Cancer Genome Atlas (TCGA) cancer types. (A) Correlation between purity estimates derived using pathology, DNA, mRNA, and microRNA (miRNA) profiles and molecular profiles (mRNA.naive = bulk mRNA abundance, mRNA.ISOpure = deconvolved mRNA abundance, miRNA.naive = bulk miRNA abundance, miRNA.ISOpure = deconvolved miRNA abundance, and CNA = bulk copy number data; deconvolved RNA profiles were generated using ISOpure). Each feature (genes for mRNA and copy number aberration [CNA] profiles, miRNAs for miRNA profiles) was correlated with tumor purity estimators (pathology, DNA, RNA, miRNA) separately. The *x*-axis represents number of purity estimators where a feature was found to be significantly correlated (Spearman’s |ρ| > 0.3, false discovery rate–adjusted *P* < .01). (B) Distribution of tumor purity estimates across 13 TCGA tumor types (4,830 tumors) using an in silico DNA-based (ASCAT) and mRNA-based (ISOpure) method. “Mean” estimate indicates combined mean of purity estimates from ASCAT and ISOpure. “Pearson’s R” indicates correlation between ASCAT and ISOpure estimates. “n” shows total number of samples with valid estimates available for both ASCAT and ISOpure.

Next, we evaluated whether pathology, DNA, mRNA, and miRNA purity estimates vary in their associations with individual genes or miRNAs and to what extent these can be overcome by using in silico deconvolution.^15^ Each of the four consensus purity estimators was individually correlated with five molecular profiles (bulk/naïve and deconvolved mRNA abundance, bulk/naïve and deconvolved miRNA abundance, and bulk copy number data; deconvolved profiles were generated using ISOpure). Here, deconvolved profiles represent signal in bulk mRNA/miRNA abundance profiles, predicted to emanate from tumor cells only, removing signal from TACs.^4,15^ Most of the features (genes’ mRNA abundance or copy number, miRNA abundance) were correlated with only one purity estimator at a time (Spearman’s |ρ| > 0.3, FDR-adjusted *P* < .01), a trend which was consistent across all five molecular profiles (Data Supplement Tables 4-8, Fig 4A). Naïve mRNA and miRNA profiles exhibited the greatest proportion of features correlated with tumor purity, which diminished after in silico deconvolution, highlighting potentially confounding TACs. With the exception of naïve miRNA profiles, purity estimates were inversely correlated with molecular profiles regardless of the underlying purity estimation profile (Data Supplement Fig 5A-F). These data suggest that the presence of genomic and transcriptomic correlates of tumor purity are likely to confound biologic and clinical interpretations.

Because DNA- and mRNA-based assays are most commonly used in cancer genomics, we asked if the purity estimates from these two analytes are comparable in other cancers. Given the strong intra-analyte correlation (Fig 1C), we considered a representative DNA-based method (ASCAT) and an mRNA-based method (ISOpure) to estimate tumor purity for an additional 12 cancer types (4,497 tumor samples) from TCGA project (Fig 4B, prostate cancer data discussed above is shown for reference only). Overall, all cancer types showed an average purity of at least 0.56. Breast cancer exhibited the lowest mean purity (0.56) and kidney renal papillary cell carcinoma the highest mean purity (0.77). Assessment of concordance between DNA- and mRNA-based estimates revealed an overall trend of poor correlation across 11 of 12 cancers (Pearson’s R = −0.27 to 0.09; Fig 4B). DNA- and mRNA-based estimates for rectum adenocarcinoma were correlated (Pearson’s R = 0.47, *P* = 3.03 × 10^−6^). However, the distribution of these two sets of estimates differed significantly (Δ_DNA−mRNA_ = −0.19, *P* = 1.09 × 10^−13^, Wilcoxon rank sum test). These data further underscore the importance of using analyte-matched purity estimates for bioinformatics analysis and subsequent interpretation.

## DISCUSSION

Herein, we provide evidence that tumor purity estimates manifest intrinsic properties of the underlying information used for purity estimation and exhibit only modest interprofile concordance. One explanation for these variations lies in the starting tissue material corresponding to the different areas of tumor specimen assessed. Pathology-based estimates are considered the gold standard. However, interpathologist variation observed in our study, as well as previous studies, suggests that there are probably some inaccuracies in these estimates because of their subjectivity/qualitative nature.^5,21^ These discrepancies may also be a result of the lack of full spatial heterogeneity of the pathologic slide. To some extent, this limitation may be overcome by increasing the observer size and spatially diverse slides per sample. However, this is often not practical in the absence of digital pathology strategies. For clinico-genomic sequencing studies requiring a minimum purity threshold for inclusion in the study, an alternative to pathology estimates is to infer purity directly from the analyte by performing low-pass DNA sequencing to filter low-purity samples.^22^

In addition to poor concordance between pathology and DNA/RNA-based tumor purity in prostate cancer, our pan-cancer data reported herein suggest that the purity estimates from DNA and mRNA profiles also show limited concordance. The concordance between purity estimators also varies depending upon the tumor type and patterns of somatic changes it exhibits (eg, DNA-based methods rely on the presence of copy number aberrations). Furthermore, previous studies have reported varying levels of concordance in purity estimates inferred from DNA- and RNA-based methods.^2,23^ For instance, Aran et al^2^ show much stronger concordance between ESTIMATE^24^ (RNA-based purity estimator) and ABSOLUTE^13^ (DNA-based purity estimator) compared with the RNA- and DNA-based methods in our study. This has significant implications because many genomic algorithms require tumor purity as an input parameter, and selection of the right algorithm for the right tumor type remains challenging. We recommend using purity estimates inferred from matched starting material. For instance, DNA analyses should be adjusted with purity estimates inferred from the DNA profiles and gene expression analyses with RNA-based purity estimates. Because purity estimates vary across methods, consensus estimates on the basis of matched analyte type may further improve purity estimates and may also overcome missing values and normalize outlier estimates. After confident purity estimates have been created, one way to account for these is to adjust bioinformatics and statistical analyses for tumor purity, as stressed in previous studies.^2,7,15^ Because bulk tumor profiles are heterogeneous compositions of tumor cells and TACs featuring complex interplay, it is crucial to interpret the clinico-genomic profiles in the context of the underlying heterogeneity.^25^ Many in silico deconvolution techniques have been developed to estimate relative abundance of different cell types,^24,26,27^ as well as techniques that explicitly generate residual transcriptomic^11,12,15,18,23^ and genomic^14^ profiles of tumor-only and stromal-only cells. Use of these residual profiles has generated optimism^4,18,23^; however, their applicability in routine bioinformatics analyses remains less popular. Herein, we recommend researchers to consider deconvolution of bulk profiles into individual component profiles (e.g., cancer and stromal profiles) to improve sensitivity and specificity of downstream analyses.^4,15^
